# Efficient Actor Recovery Paradigm for Wireless Sensor and Actor Networks

**DOI:** 10.3390/s17040858

**Published:** 2017-04-14

**Authors:** Reem K. Mahjoub, Khaled Elleithy

**Affiliations:** Department of Computer Science, University of Bridgeport, 126 Park Avenue, Bridgeport, CT 06604, USA; rmahjoub@my.bridgeport.edu

**Keywords:** wireless sensor network, wireless sensor actor network, WSN, WSAN, RSSI, latency, actor, sensor, node failure, data recovery

## Abstract

The actor nodes are the spine of wireless sensor and actor networks (WSANs) that collaborate to perform a specific task in an unverified and uneven environment. Thus, there is a possibility of high failure rate in such unfriendly scenarios due to several factors such as power consumption of devices, electronic circuit failure, software errors in nodes or physical impairment of the actor nodes and inter-actor connectivity problem. Therefore, it is extremely important to discover the failure of a cut-vertex actor and network-disjoint in order to improve the Quality-of-Service (QoS). In this paper, we propose an Efficient Actor Recovery (EAR) paradigm to guarantee the contention-free traffic-forwarding capacity. The EAR paradigm consists of a Node Monitoring and Critical Node Detection (NMCND) algorithm that monitors the activities of the nodes to determine the critical node. In addition, it replaces the critical node with backup node prior to complete node-failure which helps balancing the network performance. The packets are handled using Network Integration and Message Forwarding (NIMF) algorithm that determines the source of forwarding the packets; either from actor or sensor. This decision-making capability of the algorithm controls the packet forwarding rate to maintain the network for a longer time. Furthermore, for handling the proper routing strategy, Priority-Based Routing for Node Failure Avoidance (PRNFA) algorithm is deployed to decide the priority of the packets to be forwarded based on the significance of information available in the packet. To validate the effectiveness of the proposed EAR paradigm, the proposed algorithms were tested using OMNET++ simulation.

## 1. Introduction

**Figure 1:**
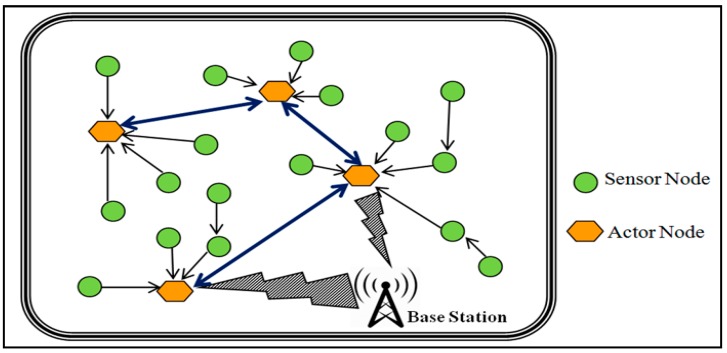
WSAN Architecture.

Wireless Sensor and Actor Networks collaborate to transmit and process information in order to perform specific tasks. WSANs comprise sensors, actors, and a base station [1] as shown in Figure 1. The sensors are responsible for sensing and transmitting the events to the actor node. In response, the actors collect, process, transmit and utilize the data, due to their high performance capability and power properties [2,3]. The communication in WSANs can be classified as: (i) sensor-actor (SA) communication; (ii) actor-actor (AA) communication; (iii) actor-sink (AS) communication and (iv) sensor to sensor (SS) communication. WSANs can further be classified into semi-automated or automated WSANs. In semi-automated WSANs, sensor nodes sense the events and forward the data to the sink node, while the sink node forward the sensed data to the proper actor in-order perform the specific action. WSANs are potentially suitable for several applications including transportation, battlefield surveillance, industry, health, environmental monitoring, smart energy grids, cloud computing, and nuclear fields. WSANs performance is affected by several factors, such as energy efficiency, transmission media, scalability, and node density. The selection of the parameters for optimization depends on the nature of the application. WSANs have an edge over the traditional Wireless Sensor Networks (WSNs) due to the high energy and low power consumption characteristics.

In WSANs, the actor nodes possess sophisticated features that increase the power capability and network usage. Thus, maintaining the inter-actor connectivity is indispensable in WSANs. The failure of an actor may cause loss of communication or a network disconnect. Thus, actors must communicate with each other to guarantee the entire network coverage and to harmonize their actions for the best response. In case of actor failure, adjacent actors should restore the process or they may be replaced by a backup actor. This solution, however, could be costly and infeasible. Alternatively, the actor can be replaced by one of its neighbor actors. Distributed localization algorithms are used to handle these situations. In this case, “hello” messages are exchanged by the neighbor actors in distributed localization algorithms despite the limitation of the network resources. Furthermore, the nodes can compute their distances and positions by exchanging the information carried in these messages. The distributed localization techniques are used for calculating the distance between the nodes, identifying the physical locations of actor nodes, reallocating the locations, and salvage of sensor or actor node failure.

Received Signal Strength Indicator (RSSI) is a range base localization technique that is applicable for determining the received signal strength including the distance between the sender and the receiver nodes. At the receiver side, RSSI technique is used to measure the signal strength [4]. Higher levels of RSSI indicate stronger signals. Some localization approaches utilize the RSSI for nodes distance measurement. In WSANs, most of the node localization and routing approaches depend on the hop count information rather than table-based routing protocols. With these motivations, this study introduces a mathematical model for determining actor forwarding capacity in WSAN using RSSI message information. The model aims at guaranteeing contention-free forwarding capacity. The paper also contributes to the literature by providing the best RSSI value to improve the traffic forwarding process. State-of-the-art research is used to provide the best node failure recovery process to extend the network lifetime. A tradeoff between the QoS improvement and reduction in the power consumption is considered.

The paper introduces three novel algorithms and optimized low latency deterministic model based on RSSI. First, Node Monitoring and Critical Node Detection algorithm checks the entire network to determine the critical node before it fails which greatly improves and balances the network performance; Second, Network Integration and Message Forwarding algorithm mostly focuses on improving the QoS by handling the packet forwarding process that has the capacity to decide the source of the forwarding packet (either from sensor node or actor node). As, this algorithm introduces much easier process to deal with packet forwarding flow. In addition, accurate packet forwarding process reduces the latency and bandwidth consumption; Third, Priority-Based Routing for Node Failure Avoidance algorithm determines the significant information available in each forwarding packet. Based on the nature of the information, the packets are routed to the next node. As, this algorithm particularly handles the redundant data prior to routing the packets; Finally, optimized RSSI model is introduced that selects the different power strengths for each beacon in order to ensure the proper delivery of the beacon to each node. This aims to reduce the latency and estimating the prediction of the node energy-level. As a result, QoS provisioning is maintained while extending the network lifetime.

## 2. Contribution and Paper Organization

The main difference between WSAN and WSN is the existence of actor nodes. Actor nodes add major enhancements and robustness to the sensor network. In WSNs detection, processing and handling are usually done using sensor nodes which make those node subject to failure and degrades the network performance, while in WSANs, sensor nodes detect events and forward their information to actor nodes. Then, the actor nodes handle the processing of information, decision making and event handling. Thus, the actor node is a major component of WSAN. The failure of an actor node can degrade the overall network performance. Furthermore, the failure of an actor node may result in a partitioning of the WSAN and may limit event detection and handling. In this paper, a new method is proposed for efficient actor recovery paradigm (EAR) which guarantees the contention-free traffic-forwarding capacity. Unlike previous studies, EAR aims to provide efficient failure detection and recovery mechanism while maintaining the Quality of service. Thus, the proposed paradigm contributes three novel algorithms and optimized low latency deterministic model based on RSSI work as follows:

- Node Monitoring and Critical Node Detection (NMCND) algorithm that monitors the activities of the nodes to determine the nodes types and distinguish critical nodes. The NMCND algorithm checks the entire network to determine the critical node during the network life time and pre-assign a backup node for each critical node; so in case the failure of critical node, this node takes place in order to improve and balances the network performance.
- Optimized RSSI model is introduced that selects the different power strengths for each beacon in order to ensure the proper delivery of the beacon to each node. This aims to reduce the latency and estimating the prediction of the node energy-level. As a result, QoS provisioning is maintained and extended the network lifetime.
- Network Integration and Message Forwarding (NIMF) improves the QoS by handling the packet forwarding process. NIMF works to reduce packet forwarding through critical nodes and enhances network lifetime. Moreover, NIMF has the capability to decide the source of the forwarded packet which enhances the packet forwarding flow. Thus, accurate packet forwarding process reduces the latency and bandwidth consumption.
- Priority-Based Routing for Node Failure Avoidance algorithm (PRNFA) handles the routing process. PRNFA analyzes and evaluates the information of the packet in order to route it to the next node. It determines the priority of the forwarded packets. In addition, PRNFA eliminates redundant data prior to routing the packets.

The remainder of the paper is organized as follows: Section 3 presents a comprehensive review of relevant literature. Section 4 formulates the problem and provides details of the optimized deterministic actor recovery system model. Section 5 provides the simulation setup and presents the analysis of our findings. Finally, the summary of the paper is provided by the conclusions in Section 5.

## 3. Related Work

In this section, the salient features of relevant related approaches are discussed. WSANs are difficult to deploy, even though WSANs are known to improve the overall network performance. One shortcoming of WSANs is that these networks adversely are affected by inadequate positioning, power restraints, and routing limitations. To avoid these issues, the sensor and actor nodes should be deployed randomly or at fixed position based on the application requirements. Actor nodes can either be mobile or static. Hence, the node mobility improves the network performance metrics such as coverage, connectivity and lifetime [5]. A number of localization techniques were studied and introduced for WSANs. Some techniques focus on nodes positioning are provided in [6], while some other studies focus on failure node recovery process [7,8,9,10,11,12,13,14,15].

WSANs are mainly deployed in harsh areas, and are supposed to support long network lifetime. As we mentioned earlier, actor nodes are very essential nodes in WSAN. Thus, it is essential for WSAN algorithms to support not only actor node deployment and actor mobility, but they should be robust enough to provide failure detection and self-healing network recovery.

Some works presented in the literature manage to handle the actor node deployment and mobility [16,17,18,19,20,21,22,23]. Nevertheless, they do not provide solutions in the case of the occurrence of actor node failure. The study in [20] presents a framework for real-time data report and task execution (RTRE) which aims to achieve event assignment through multi-actor coordination and data collected coordination between sensor and actors real-time sensor-actuator data collection. The authors of [23] present a self-organizing mobility control in WSANs based on virtual electrostatic interactions which aim to enhance actor node deployment and control actors’ mobility. The main purpose of the previously mentioned techniques is to manage actor mobility during deployment or during event handling. Meanwhile, actor mobility features may cause continuous changes to the network topology. Thus, an actor may go outside a specific region range and is then defined as failed.

In WSANs, actor failure can be due to their limited power, mobility, or topology changes. The mobility feature can cause actors to go outside the communication range. Moreover, network topology may be affected by such behavior. Effective topology management techniques should be implemented. Several proposed mechanisms where introduced in order to manage network failure concerning topology management [5,11,13,24,25,26,27,28,29,30,31,32].

Fault detection mechanisms are classified into proactive and reactive methods. In proactive methods, fault and restoration mechanisms are addressed during the network setup. Some mechanisms implement fault tolerance topologies in the network setup while others use redundant and backup nodes to ensure fault tolerance [33]. On the other hand, a reactive scheme pursues to utilize the network resources and performs recovery through node repositioning. Reactive schemes require network monitoring in order to maintain node status. Network status, recovery algorithms, and recovery scope are important factors in reactive schemes. Reactive recovery algorithms are classified into centralized algorithms or distributed algorithm and have been widely used in the literature [26,29,30,32,33,34,35,36,37,38]. Scope of recovery refers to how many nodes are involved in the recovery. Some mechanisms require a single node [35,36] while others identify a block of nodes for the recovery process [10,39].

Moreover, actor fault impact can vary depending on the node’s importance and type. Some fault management detection and recovery procedures classify the actor nodes into critical nodes and non-critical nodes [10,24,37]. A critical node refers to a node which failure causes network partitioning. Most algorithms define the critical nodes using 2-hop message exchange information [8,36]. On the other hand, a study conducted by Imran et al. [40], used 1-hop message exchange to identify the critical actors. This is performed by calculating the distance from the actor to its adjacent nodes. If the distance is less than the neighbor’s communication range, the actor is defined as non-critical; otherwise, the actor is defined as critical.

The Distributed Recovery from Network Partitioning in Movable (DRNPM) [40] and Actor Positioning with Minimal Movement (APMM) [7], in Wireless Sensor and Actor Networks approaches were introduced for node recovery. These studies applied pre-assigned backup procedures to recover the actor node failure. However, the approaches fall short in addressing the energy efficiency during backup node selection.

In [35] a two-hop actor node failure recovery algorithm is introduced in which, an adjacent actor and best candidate are selected to handle the failure node. However, this approach suffers due to the overhead. Haider et al. [36] introduced Nearest Non-critical Neighbor (NNN) algorithm that attempts to achieve inter-actor connectivity caused by network splitting. The proposed algorithm uses a localized and distributed approach. When the neighbors of a critical actor detect the failed actor, they initiate the reinstatement process. This process consists of the replacement of the critical node with the nearest non-critical actor to control any further splitting overhead. The overhead may occur when a critical actor movement is selected for node replacement. Distinguishing between critical and non-critical actor nodes favors the NNN procedure to have slighter recovery scope in comparison to [35]. However, the network is adversely affected due to spilt transposition overhead. A new energy efficient recovery method for recovery of lost connectivity (RLC) based on two-point crossover genetic algorithm (GA) is proposed to reconnect the partitioned network to discover the actor node failure process [41].

A study that handles the tolerating simultaneous failures (TSF) in WSANs is proposed by [42]. In this study, TSF is based on the ranking of the network nodes pertinent to a pre-assigned root actor. Ranking uses a tree that helps the coordination process among the nodes. The nodes are virtually grouped in order to minimize the recovery overhead. Cluster-based node failure algorithms were proposed by [43,44] that are based on 1-hop node failure recovery process.

Furthermore, the route duration improvement (RDI) algorithm based on decision tree is incorporated in the reactive routing protocol to support WSANs [45]. This aims to select the most long-lived routers. The decision tree supports the node mobility. The improved flooding control is used to improve the route performance and reduce the overheads, whereas, power consumption caused by control packets is not handled.

Similarly, recovering from node failure (RNF) based on the Least-Disruptive Topology Repair (LeDiR) algorithm is used to handle actor failure/recovery. The RNF handles the autonomous transposition for the subset of the actor nodes to restore connectivity [10]. The LeDiR algorithm depends on the local view of the actor node to develop a recovery plan that rearranges the least number of nodes and confirms that no path between any pair of nodes is protracted. In addition, LeDiR attempts to detect and manage cut-vertex node failure and perform recovery using path discovery and routing information. In the case of node failure in LeDiR, neighboring nodes will re-compute their routing tables and drive their enrollment decisions for the recovery process. In response to the failure of a critical node, the neighbor containing the smallest block replaces this node. LeDiR assumes that each node calculates the shortest path to every other node and stores this information in its routing table. In case node failure occurs, the 1-hop neighbors identify if the node is critical or non-critical using the shortest path routing table. Then, the smallest block is identified. Within the smallest block, a neighbor of a failed node is chosen as the candidate node (CN) to replace the failed node. If more than one neighbor node is part of the smallest block, the neighbor nearest the failed actor is chosen to manage the block movement. The RNF aims to control existing route discovery activities in the network and enforce no extra pre-failure communication overhead. However, RNF consumes an additional power. Moreover, large number of nodes are involved in the recovery process which leads to more topology changes; thus, network is subject to extended failure causing a cascade recovery.

Grid-based approaches are introduced in many WSANs. As one of the interesting grid-based approaches, an actor-supported repositioning approach supports the single static actor. Each grid consists of single static actor, in addition to several sensor nodes that sense the events. Static actors are responsible to obtain the information of sensors location along with grid information. Hence, the grid information collects the region information. Once an event occurs, the sensors nodes forward sensed data to the static actor node. The overhead of the approach increases when using single static actor node for multiple grid region monitoring [46]. This problem surges when multiple reporting regions instantaneously report to the same static node [14,30,47,48,49].

The distributed prioritized connectivity restoration algorithm (DPCRA) [11] is introduced to cover the partitions and reinstate the node connectivity by using small number of nodes. The algorithm aims to identify the negative effect of the actor on the partitions. Repairing processes are done locally while storing minimum information in each node. The main focus of the work is to use multiple backup nodes for the partitioned recovery. Nevertheless, the algorithm fails to address backup node selection criteria which leads that those nodes may have higher probability of failure. Thus, this can affect the overall network performance, especially energy consumption, which leads to higher probability of node failure throughout the network.

The actor critical recovery (ACR) algorithm is proposed for efficient resource utilization [7]. The algorithm aims to minimize the delay and determine the primary backup node to satisfy application requirement. In this study, Akkaya et al. proposed the Distributed Partition Detection and Recovery algorithm (DPDR) to handle cut-vertex node failure recovery. The main objective of the work is to minimize node movement distance during the recovery process. Cut-vertex node determination is done using Depth First Search (DFS). DPDR assigns a failure handler (FH) node for each cut-vertex. FH is responsible for the network recovery when the cut-vertex failure occur. FH chooses to replace the failed node with the node which has the closet distance to the failed node. The main drawbacks of DPDR are the involved communication and calculation overheads, and FH recovery assignment criteria.

The Advanced-self-healing Connectivity Recovery Algorithm (ACRA) is introduced to recover failed actors. The ACRA determines the nature of the actor whether it is a cut vertex or node-connectivity actor [13]. This approach applies a depth-first search algorithm to determine the nature of the actor. For handling suddenly failed nodes, the minimal block backup nodes are used until the network is restored. The actor node with high transmission power and higher coverage area is selected and connectivity is recovered. This type of process consumes more energy since cluster head and actor node has to be selected in case of their absence in the network. The algorithm is based on two point crossover Genetic Algorithm (GA) to reconnect the partitioned network. Sensor and actor nodes are scattered randomly in the area of interest and form clusters. In this network, all the nodes are equipped with a failure detection system and are able to detect the failure of cut-vertex actor nodes by using the shared stored information of their 1-hop neighbors. Whenever a cut-vertex failure is detected, the neighbor CHs broadcast Recovery messages to all their neighboring nodes toward the sink node until the next actor node or the next CH is found for lost connectivity. Recovery Phase is executed by finding a stable sensor with high transmission power and higher coverage. The stable sensor CHs (as per the GA-based criterion) among their neighbor nodes is the bridging router for connecting the disjoint network. Even though actor nodes include higher transmission power, the process still consumes more energy; the algorithm consumes energy because of the use of clustering. Moreover, sensor involvement in the recovery process impacts the overall network lifetime and performance due to the sensors’ limitations.

The Partitioning detection and Connectivity Restoration (PCR) algorithm is introduced to endure critical actor failure [50]. The PCR regulates critical/non-critical actors using localized information and replaces each critical node with a suitable backup. The pre-designated backup process determines the failure if it is primary actor node and starts the post-failure recovery process that includes coordinated multi-actor relocation. The author constructed the formal specification of PCR using Z notation. An update of the selection of the actor’s polling points is proposed in [51]. The update selection process involves the residual energy and the locations of the nodes. The approach dynamically generates the multi-hop routing trees used by the polling points in order to balance energy consumption of the node to prolong the network lifetime. However, the paper focused on sensor nodes and also did not address the actual node failure recovery process.

All existing approaches either attempt to recover the failure actor or try to reduce the overhead [8,9,30,31,32,33,34,35,38,52,53,54]. We conclude that existing approaches have attempted to replace the critical node with another backup node, but they fail to maintain the QoS parameters and energy consumption. For guaranteeing QoS in our EAR proposed algorithm, the Node Monitoring and Critical Node Detection algorithm (NMCND) monitors the activities of the nodes to determine the nodes’ types to distinguish critical nodes. Additionally, our proposed approach not only determines the critical nodes, but handles the packet forwarding process when a primary node fails. To handle packet forwarding, the Network Integration and Message Forwarding (NIMF) algorithm is introduced. In addition, the Process-Based Routing for Node Failure Avoidance algorithm (PRNFA) is developed to handle the routing process and to eliminate routing process of the redundant packets to other nodes in order to avoid the network congestion and reducing the latency. Therefore, the goal of this work is to improve the recovery node process while maintaining the QoS provisioning and power efficiency.

## 4. Problem Statement and Optimized Deterministic Actor Recovery System Model

### 4.1. Problem Formulation

WSANs comprise actors with powerful resources and sensor nodes with limited computation, power, and communication capabilities. The sensors and actors in WSANs collaborate together to monitor and respond to the surrounding world. WSANs can be applied to a wide range of applications, like health or environmental monitoring, chemical attack detection, battlefield surveillance, space missions, intrusion detection, etc. However, WSANs are greatly affected due to environmental changes, frequent changes in event-detection and actor failure processes. The failure of an actor node can result in partitioning of the WSAN and may limit event detection and handling. Actors may fail due to hardware failure, attacks, energy depletion, or communication link issues. Sensor node failure may cause loss of event detection of the assigned environment covered by the sensor. The probability of the actor failure is less than that of sensor failure and can be controlled through the relocation of mobile nodes due to their powerful characteristics; however, actor failure can cause more damage than sensor failure. Actor failure can cause a loss of coordination and connectivity between nodes, limitation in event handling, and can lead to a disjoint WSAN.

**Figure 2:**
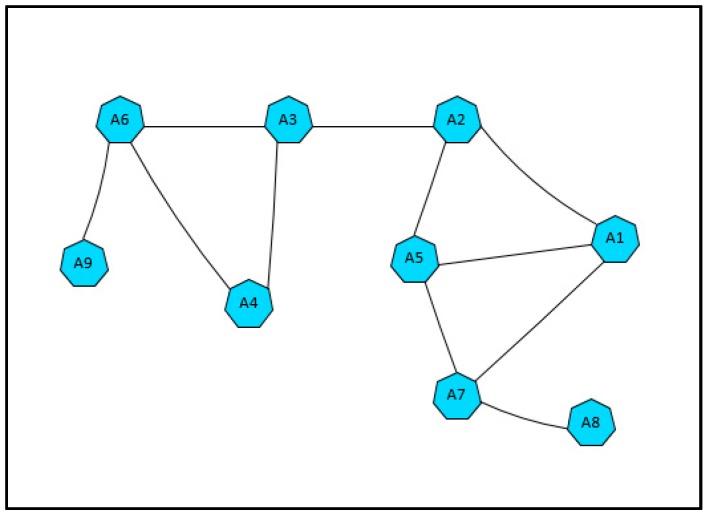
Critical actor node in WSAN.

The actor failure occurrence is very critical as it degrades the network performance. The failure of a critical actor may cause high impact to the whole network. Critical actor nodes refer to actors which their failure cause network partitioning. Figure 2 illustrates the concept of critical actor nodes. Assume that actor A3 failed. Its failure will cause the network to disjoint. Thus, A3 is a critical node. Actor nodes A2, A6, and A7 are critical nodes as well.

Most of the existing approaches attempted to replace the critical node with another backup node, but they failed to maintain the QoS parameters and energy consumption. For instance RNF manage to handle failure by moving a small block of neighbor actors toward the failed node in order to recover the communication among them. Even though this manages the recovery of the network but it enlarges the recovery scope and cascade relocation. Such behavior should be eliminated in recovery algorithms. In addition, due to the fact that WSANs are deployed in harsh areas and require long term monitoring/acting process, proposed methods should offer robust self-healing failure detection/ recovery techniques which ensure that the network lifetime is maximized as much as possible while maintaining QoS.

In WSANs, the nodes track their neighbors by using heart beat messages to avoid any possible interruption. Moreover, algorithms are used to define the critical nodes using 1-hop or 2-hop message exchange information [8,36,40]. In addition, they identify the actor node failure by the interaction of those heartbeat messages with this particular actor node. Thus, there is possibility of interruption due to losing the trail of heart beat messages. Monitoring actor failure detection using 2-hop neighbor list is efficient once it is combined with QoS measurement capabilities, i.e., packet delivery and forwarding techniques should support efficient packet handling and forwarding. Also, we should minimize the through critical actor nodes. Thus, in our proposed model, we assume that each actor node stores the information up to 2-hops to keep the extended trail information. This helps determine the forwarding capability of the actor nodes. The model aims to ensure the contention-free forwarding capability that minimizes the loss of packets in case of node failure. To determine the actor’s forwarding capability, each actor conveys the group of beacon messages using different power strengths. Furthermore, the neighbor of each actor listens and returns the value in response. After the neighbor actor receives the message, it starts calculating its RSSI value and sends it back to the sender actor. The RSSI model is used to calculate the distance. It has also been combined with further techniques for better accuracy and to find the relative error. Equations (1)–(5) illustrate applying RSSI in actor nodes [55]. RSSI can also be used to determine the link quality measurement in wireless sensor networks [56].The RSSI shows the relationship between the received energy of the wireless signals and transmitted energy and the required distance among the actor-sensor nodes. This process helps determine failure node recovery process given in Definition 1. The relationship is given by Equation (1):

(1) $$E_{r}=E_{t}\times {\left(\frac{1}{r}\right)}^{\beta }\;$$

where $E_{r}$: Received energy of wireless signals, $E_{t}$: transmitted energy of wireless signals, r: Distance between forwarding and receiving node, and β: Path loss transmission factor whose value depends on the environment.

Taking the logarithm of Equation (1) provides:

(2) $$10\log E_{r}=10\log E_{t}-\;10\beta \log r$$

where 10logE: Description of energy that could be converted into dBm.

Therefore, Equation (2) can be converted to its dBm form as:

(3) $$E_{r}\left(dBm\right)=\gamma -\;10\beta \log r$$

where γ: transmission parameter.

Here, γ and β represent the relationship between the strength of the received signals and the distance of the signal transmission among sensor-to-sensor, actor-to-sensor or actor-to-actor.

RSSI propagation models cover free-space model, log-normal shadow model and ground bidirectional [20]. In this study free space model is used due to following conditions.

▪ The transmission distance is larger than carrier wavelength and antenna size.
▪ There is no obstacle between forwarding actor and either receiving actor or sensor.

The transmission energy of the wireless signals and the energy of the received signals of sensor nodes located at distance of ‘*r*’ can be obtained by Equations (4) and (5):

(4) $$\frac{E_{t\;}A_{gt}A_{gr}\lambda^{2}}{{\left(4\pi \right)}^{2}r^{2}\omega }$$

where λ: 1/Frequency of the actor node, $A_{gt}\;\&\;A_{gr}$: An antenna gains, ω: failure factor of the actor and r: distance of the node:

(5) $$E\omega \left(dB\right)=10\log\frac{A_{\mathrm{gt}}}{A_{\mathrm{gr}}}\;=-10\;\log\left[\frac{\lambda^{2}}{{\left(4\pi \right)}^{2}r^{2}}\right]\;$$

Equation (5) represents the signal attenuation using a logarithmic expression. Assume a field with k actor nodes $a_{1}$, $a_{2}$, $a_{3}$,..., $a_{k}$. The coordinates of the actor nodes are $\left(p_{i},\;q_{i}\right)$ for *i* = 1, 2,…, *k*. The actor nodes transmit the information regarding their location with their signal strength to the sensor nodes {$s_{1},{\;s}_{2},\;.\;.\;.,{\;s}_{n}$}. The locations of the sensor nodes are unknown. The estimated distances of the actor nodes are calculated from the received signals. In the proposed model, the actor nodes broadcast signals to all sensors. The actor nodes are also responsible for estimating the distances between them and sensor nodes. Let $a_{i}$ be an actor node located at $\left(y_{i},z_{i}\right)$ and sensor node is located at (y, z). Focusing on the relative error $‘r_{e}’$ relating to $a_{i}$, suppose that the actor node reads a distance $r_{i}$, but the correct distance is $r_{j}$. Therefore, the relative error can be obtained by Equation (6):

(6) $$r_{e}=\frac{r_{i}}{r_{j}}-1\in \left(-1,\;+\infty \right)\;$$

The relation between actual distance and the measured distance can be obtained by Equation (7):

(7) $$r_{i}=r_{j}10\frac{\pi }{10\beta }-1\;$$

which can be reduced to Equation (8):

(8) $$r_{e}=10\frac{\pi }{10\beta }-1\;$$

The probability distribution of the location of the actor node based on beacon messages is described in the following definition.

**Definition** **1.** *Let* $a_{i}$ *be an actor node located at* $\left(y_{i},z_{i}\right)$ *that sends information to a sensor using RSSI model with standard deviation* ‘σ’ *and path loss* ‘β’.

*Let* $r_{i}$ *be the calculated distance from the actor node* $a_{i}$ *at the sensor node. The probability density function for correct location* (y,z) *of the sensor node is obtained by Equation (9):*

(9) $$\left\{E_{Y,Z}^{\left(i\right)}\left(Y,Z\right)\frac{10\beta \exp\left(-{\left(\log\beta \log\frac{r_{i}}{\sqrt{{\left(y-y_{i}\right)}^{2}+\left(z-z_{i}\right){\;}^{2}}}\right)}^{2}/2\sigma^{2}\right)}{2\sigma^{2}\sqrt{2\pi \;\log\left(10\right){\left({\left(y-y_{i}\right)}^{2}+\left(z-z_{i}\right){\;}^{2}\right)}^{2}}}\right\}$$

*Probability distribution can be simplified due to an actor* $a_{i}$ *with Equation (10):*

(10) $$E_{Y,Z}^{\left(i\right)}\left(Y,Z\right)=\Psi_{a_{i}}\left(y,z\right)\;$$

This can further be extended by using finite set of actors $A=\{a_{1}$*,* $a_{2}$*,* $a_{3}$,…, $a_{k}$} that produces Definition 2.

**Definition** **2.** *Let* $A=\{a_{1}$*,* $a_{2}$*,* $a_{3}$,..., $a_{k}$*} be the set of the actors sending information to the set of sensor nodes using RSSI model with path loss exponent* ‘β’.

*If the calculated distance from the actor node* $a_{i}$ *at the sensor nodes S* = *{*$s_{1},\;s_{12},\;.\;.\;.\;,\;s_{n}$*}, then the probability density function of correct location* (y,z) *of the sensor nodes can be obtained by:*

(11) $$\left\{\Psi \left(A\right)\left(y,z\right)=\frac{\sum_{i=0}^{k}\Psi_{a_{i}}\;\left(y,z\right)}{\int_{-\infty }^{+\infty }\int_{-\infty }^{+\infty }\left(\sum_{i=0}^{k}\Psi_{a_{i}}\left(y,z\right)dzdy\right)}\right\}\;$$

*where* $\Psi a_{i}\left(y,z\right)$*: probability distribution because of an actor* $a_{i}$*.*

**Definition** **3.** *Assume an actor node *a_i_* reads a sample distance* $R=\{r_{1}$*,* $r_{2},\;r_{3},\;.\;.\;.,\;r_{n}\}$ *using beacon messages* $B=\{b_{1}$*,* $b_{2},\;b_{3},\;.\;.\;.,\;b_{n}\}$ *that is modeled with RSSI with path loss* β *and standard deviation* ‘σ’.

*If ‘R’ provides the mean sample distances and* $\sigma_{i}$ *is the mean standard deviation, then the square of the actual distance from actor to sensor using beacons can be determined as:*

(12) $$R=\frac{R^{4}}{R^{2}+\sigma_{i}^{2}}\;$$

*Furthermore, square standard deviation can be found as:*

(13) $${\sigma_{i}}^{2}=\frac{100\beta^{2}}{\log^{2}\left(10\right)}\;\log{\left(1+\frac{\sigma }{R}\right)}^{2}$$

The definition shows that the actual distance is greatly dependent on the distribution of the measured ranges.

Hence, our proposed formulas for RSSI-based wireless node location are optimized and modified. They are different from the original RSSI-based formulas. We focused particularly on the energy consumed for transmission and receiving the data including determining the distance between actor-sensor nodes and error rate for finding location of the node that helps identifying the accurate position of the deployed actor nodes for events. Thus, the previous model is used by our proposed algorithm in order to identify the node locations during deployment in addition to during the network lifetime.

### 4.2. Optimized Deterministic Actor Recovery System Model

**Figure 3:**
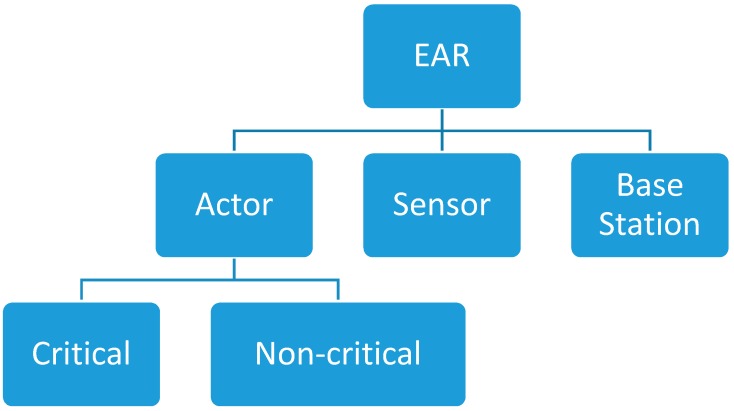
Nodes Types in EAR.

The network consists of multiple actors and sensor nodes that are structured with the hierarchical structure of the nodes. The hierarchical structure of the nodes provides an efficient, fast and logical packet forwarding patterns. It also determines the features of all nodes connected with WSANs. Another advantage of the hierarchical structure is that it helps to start with little multiplexing process for intra-domain routing. As the packets travel further from the source node the model helps to develop higher degree of multiplexing. The nodes of different categories in WSAN as depicted in Figure 3 possess the assorted nodes types. The network aims to use the resources efficiently for each packet forwarded by an actor node. In addition, it reduces the latency while keeping the network more stable.

**Definition** **4.** *Critical actor node is the actor node which its failure cause network partitioning*.

**Definition** **5.** *Non-critical nodes (NCNs) are regular actor nodes.*

**Definition** **6.** *Cut Vertex Nodes (CVNs) are nodes which have a cut-vertex link with a critical node, i.e., neighbors of critical node*.

**Definition** **7.** *The Critical Backup Nodes (CBNs) are actor nodes that are assigned to be the backup nodes for a critical node*.

The EAR consists of actor nodes, sensor nodes, and base station. Actor node can be critical or non-critical. Critical actor node is the actor node which its failure causes network partitioning. Non-critical nodes are regular actor node. Sensors node are used to monitor the network for event detection.

In this topology, Cut Vertex Nodes (CVNs) are responsible for the removal of the paths that lead to the critical nodes. When the actor node becomes a critical node, then it is necessary to redirect the traffic of the neighbor nodes to the non-critical nodes. Thus, this task is done by removing the vertex (a path leading to critical nodes) and redirecting the traffic, as it further helps improving the throughput performance and reduces the latency. The Critical Backup Nodes (CBNs) replace the actor nodes when the actor nodes become the critical nodes. We assume that the number of critical backup nodes are more than actor nodes in the network. If all the actor nodes become critical nodes, then replacement should be much easier to avoid any kind of interruption or data loss. There could a possibility of disconnecting the direct communication links of the actor nodes towards the backup nodes when the actor nodes start moving. Thus, we also assume that the links of the actor nodes lead to the backup nodes are always stable despite the mobility. Therefore, there is a high possibility to easily replace the critical nodes with CVNs. The actor node has a privilege to collect the data from event-monitoring nodes (sensor nodes), then it forwards the packets to either base station or sensor /actor nodes in the network. On the other hand, the least degree Non Critical Nodes (NCNs) are preferred to be labeled as backup nodes for event-monitoring nodes.

A neighbor node that is available in Node Distance range (ND) has a similar Cut Vertex Node Distance (CVND). This helps reduce the recovery time and overhead which is important for resource-constrained mission-critical applications.

In the network, each actor node maintains its 2-hop neighbors’ information using heartbeat messages. This information helps to maintain the network state, defining critical actor; as well as assigning backup node for the critical actor. Each actor node saves its neighbors information which includes node ID, RSSI value, number of neighbors which is denoted by the degree of node, node criticality (critical actor/non-critical actor), and node distance. Once a critical node is detected, the backup assignment process is executed in-order to assign a backup node for this critical node. The 2-hop node information is used in the process of backup node selection. Depending on RSSI value (extracted from mathematical model), the non-critical node with the least node degree is preferred to be chosen as a backup node. In case there is more than one neighbor with the same node degree, the neighbor with the least distance is preferred. For each critical actor, a pre-assigned backup actor node is selected which is called Critical Backup Nodes CBN. Consequently, CBN monitors its critical node through heartbeats messages and handles the backup process in case the failure of its critical node. Missing a number of successive heartbeats messages at CBN indicates the failure of the primary.

The topology of the WSAN can be changed during the network lifetime due to the mobility feature of actors, actor node failure, or event handling. Backup nodes are subject to failure as well. Therefore, there are primary backup nodes that select other backup nodes in case of primary backup nodes fail or move beyond the range of ND. To ensure the effectiveness and availability of backup node, we introduce novel backup node selection process in case of primary backup is either failed or in critical condition given in Algorithm 1. Algorithm 1 shows the backup selection process. In this process, the condition of primary backup $P_{b}$ node is checked. If a primary backup node is in critical condition or ready to move, then a secondary backup node $S_{b}$ is chosen. However, if a secondary backup node $S_{b}$ is in critical condition or ready to move, then tertiary backup node *T_b_* is notified to play a role as primary backup node. If the tertiary backup node is in critical condition then the backup assignment algorithm executes and a backup node is assigned.

Since actor nodes are rich-resource nodes, we further use the least square approximation formulation to identify the power strength. This involves little computational overhead which can be easily attuned in an actor so that we identify the power strength because there is a probability of the node to mislay the power at some particular time.

**Algorithm 1:** Backup Node Selection Process$P_{b}$: Primary Backup; $Pb_{C}$: Critical Primary Backup; $Pb_{m}$: Moving Primary Backup; $S_{b}$: Secondary Backup; $Sb_{C}$: Critical Secondary Backup; $Sb_{m}$: Moving Secondary Backup; $T_{b}$: Tertiary Backup. **Input:** ($P_{b}$)**Output:** ($\;S_{b},\;T_{b}$)**If** $P_{b}$ =$\;Pb_{C}$ || $Pb_{m}$//The condition of primary backup node is checked.Notify $S_{b}$ and Set ($S_{b}$,$\;P_{b})$//Secondary backup node is assigned as primary backup node**end if****If** $S_{b}$ == $Sb_{C}$ || $Sb_{m}$//The condition of Secondary backup node is checked.Notify $S_{b}$ and Set ($S_{b}$,$\;P_{b})$//Tertiary backup node is notified to play a role as primary backup node**end if**

We use node monitoring process to monitor the node pre-failure causes, the post-failure causes and allocates the recovery options. Once each critical actor node picks a suitable backup, then it is informed through regular heartbeat messages (Special signals are sent to neighbor node to play a role as backup node for critical node). Furthermore, the pre-designated backup initiates monitoring its primary actor node through heartbeats. If a number of consecutive heartbeats are missed from the primary actor, then it notifies that the primary actor failed. Thus, a backup node replacement process is started as given in algorithm 1. Before substantiating the post failure process, we must ensure that connection is not interrupted because of the network. In addition, any redundant action of the network must be controlled to avoid any possible increase of the network overhead. The pre-failure backup node process is given in Algorithm 2.

**Algorithm 2:** Node Monitoring and Critical Node Detection (NMCND) process{
$F_{pr}$: Pre-Failure; $F_{po}$: Post-Failure; $R_{pr}$: Recovery process; $N_{c}$: Critical node; $N_{b}$: Backup node; $A_{p}$: Primary actor; $M_{hb}$: Message heartbeat; $N_{s}$: Sink node} **Input: {**$N_{c}$;$\;N_{b}$;$\;A_{p}$}**Output**: {$R_{pr}$; $F_{pr}$;$\;F_{po}$}**Set** $A_{p}$//Number of actor nodes are set as primary actors in the network$N_{s}$ broadcasts $M_{hb}$//Sink node broadcasts the message to all primary actor nodes**If** $A_{p}$ ≠ $F_{pr}$ then//Determine $N_{c}$//Initiate critical node discovery process**If** ∀ $A_{p}$: $A_{p}$ ∈ $N_{c}$ then$N_{b}$ assigns $N_{c}$**Set** $N_{c}$ = $M_{hb}$**end if****If** $M_{hb}$ NotDelivered $N_{c}$ then**Set** $N_{b}$ for data delivery**end if****Process** $A_{p}$ ∃ $R_{pr}$//Primary actor node recovery process is conducted**end if**

As shown in Algorithm 2, $A_{p}$ represents the number of actor nodes in the network. Then, the sink node $N_{s}$ broadcasts the message to all primary actor nodes to determine pre-failure actor nodes. If primary actor is not identified as pre-failure, then the process of determining the critical actor node will be started in order to choose the backup node. Next, the critical node discovery process is initiated. For each primary node, if the primary node is found as critical node then this node will be defined as critical node $N_{c}$ and a backup node $N_{c}$ selection is assigned. The critical node will broadcast a message to its neighbors which includes the information of its backup node. This information is stored by the neighbors and it is used when starting the network recovery process in case the neighbors detect the failure of their critical actor neighbor. If consecutive heartbeat messages are not received from the critical node, then back node starts replacing the critical node to avoid any kind of packet-forwarding delay. In addition, neighbors of the critical node will use stored information to communicate with the backup node in order to restore connectivity. In conclusion, the recovery process is conducted.

**Figure 4:**
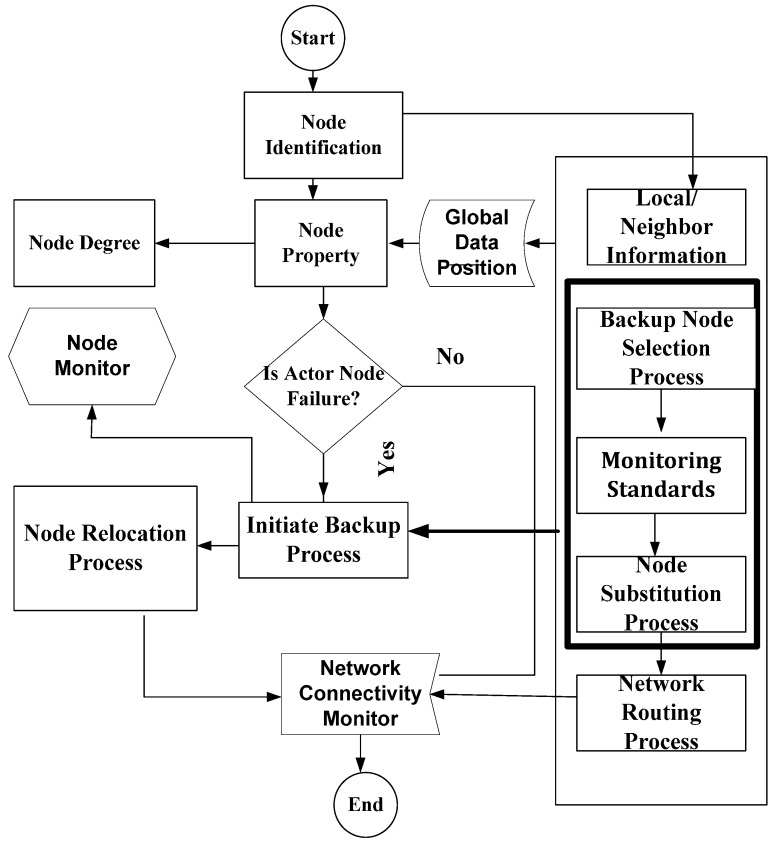
Efficient Actor Recovery system model implementation.

After the node monitoring process is executed, we proceed further with checking the backup assignment and the critical back up assignment of the nodes. This allows to maintain the network connectivity without generating any disjoint procedure of the network. The possibility of the recovery depends on the cut vertex node. If the backup is a non-critical node, then it simply substitutes the primary actor node, and the recovery process is initiated to confirm the backup actor node. If the backup is also a critical node, then a cut vertex node replacement is completed. The pre-assigned backup actor node instantly activates a recovery process once it senses the failure of its primary actor node. The complete node monitoring including failure, recovery and replacement processes are depicted in Figure 4. In complete node monitoring process, first, the node identification process is initiated. The node is identified based on local neighbor information (LNI) that involves global data position, node property and node degree. The critical node selection process is decided using Algorithms 1 and 2. Once a critical node is identified, it will be assigned a backup node; second, the backup node selection process is started if an actor node fails. The selection process is decided based on monitoring the algorithms explained earlier. Once the backup node selection process is complete, then the backup process starts working in case of node failure. If an actor node does not fail, then the node connectivity monitoring process is started and routing connectivity metrics are checked to ensure whether there is no problem of the router.

After completion of the actor node failure and node assignment processes, the actor nodes should be linked to forward the collected data to the base station. In response, the base station sends its identity (ID) using network integration message (NIM). When an actor node receives NIM from the base station, it saves the destination address of the base station for packet forwarding (PF). Subsequently, NIM is broadcasted in the network among all the actors. At least one actor node is within the range of the base station to avoid any bottlenecks. Otherwise, the base station receives the data through sensor nodes that could be the cause of packet delay and loss. The actor node saves the information of the first actor node from which it receives NIM to use PF process and further forwards NIM with its ID. If an actor gets NIM from multiple actors, then it stores the identity of additional actors in the buffer list. Identity of the saved actors is used in case of topology changes due to mobility or node failure. The detailed process of an actor node that receives NIM is presented in Algorithm 3.

**Algorithm 3:** Network Integration and Message Forwarding Process { $B_{s}$: Base Station; NIM: Network integration message; PF: packet forwarding; $N_{id}$: Node identity; $N_{a}$: Actor node; $N_{b}$: Node buffer}**Input: {**$\;B_{s}$,$\;N_{a}$ **}****Output**: {NIM, PF}**Set** NIM//Network integration message is set to interconnect the entire network**Set** PF//it saves the destination address of the Base station for packet forwarding**If** PF = $B_{s}$ then//If a base station is saved as the destination addressDecline NIM//If base station is found, then NIM is declined **else if** NIM $\in B_{s}$//**Set** PF= $N_{id}$// Data forwarding packet is given ID transmitted to Base stationTransmit NIM **else if** NIM ∈ $N_{a}$ then//It will be considered that NIM is forwarded by an actor node**end if****end else****If** PF ∈ $N_{a}$ //If it is validated that data packet is forwarded by an actor node, t$N_{a}$ stores $N_{id}$ into $N_{b}$//When actor node receives NIM from multiple actorselse **Set** PF = $N_{id}$//Data forwarding packet is given ID Transmit NIM//NIM is transmitted by an actor node**End if****end else****else if** NIM ∈ $B_{s}$ then//NIM message is broadcasted by the Base station.**If** PF ∈ $N_{a}$ then//If data forwarding packets is from an actorDecline NIM//If actor node is found, then NIM is declined**else** PF = $N_{id}$ ∈ $N_{a}$//Each forwarded data packet is given identify from an actorTransmit NIM//network integration message is transmitted by an actor node**end else****end else****end if**

Our protocol applies a simple algorithm to process the NIM. The actor node first transmits NIM to its higher hop neighbor actors/sensors. When it gets the first NIM from the higher hop actor/sensor, then it forwards to its lower-hop neighbor actors/sensors to ensure the transmission of NIMs in the entire network. All other NIMs are then dropped by the actor nodes. Therefore, if each actor is ensured to be in the communication range of at least one actor, then the NIMs should not require to be managed at sensor nodes.

Let us assume that an actor node transmits the number of bits in each packet $‘P_{r}’$ that uses encoding mechanism to reduce the complexity of each packet. The sensor nodes monitor the events which check its contribution table that specify the important events. If events are of the significant interest, then the sensor nodes generate the packets and forward to the actor node. The complete process of monitoring the events and forwarding the routing of the data packets is given in Algorithm 4.

**Algorithm 4:** Priority-Based Routing for Node Failure Avoidance Process **{**$\;P_{r}$: Packet rate; $N_{a}$: Actor node; $S_{i}$: Significant; $F_{i}$: First interest, $N_{sc}$: Sharing capacity of node; P: Packet; $N_{c}$ : remaining output capacity of the node; $E_{r}$: Efficient packet rate; $F_{i}$: Flag of interest; $F_{u}$: Flag of uninterested;$\;N_{f}$: Node failure}**Input:** {$P_{r}$, P }**Output:** {$\;N_{f},\;F_{i}$, $F_{u}$ }**If** P received by $N_{a}$//If actor node receives the packet**If** P ∈ $S_{i}$ then//If the received packet is of significant interestSet $F_{i}$//If condition in step-4 is satisfied, received first packet is considered as significant of interest.Set $N_{sc}$ +1 & decrease $N_{c}$ $by\;P_{r}$ //Sharing capacity of the node is increased**end if****end if****If** $N_{c}$ > 0 then//Determine the power of node Forward  P //Received packet is forwardedSet $F_{i}$//Flag of interest is set in the buffer**Else if** $N_{c}$ < 0 thenProcess $P_{r}$ > $E_{r}$ & P ∈ $F_{u}$//Showing that packet rate is higher than efficient packet rateIncrease $N_{c}$ $by\;P_{r}$/ remaining capacity of the actor node is increasedReduce $N_{f}$//When capacity of the node is increased, less possibility of node failure**end else****end if**

## 5. Simulation Setup and Experimental Results

There are two processes running throughout the network’s deployment and monitoring, the underlying process obtains individual node properties while the second monitors the network consistency. Our goal is to prolong the network lifetime while maintaining the minimum overhead and determining the nodes’ failure causes. We have implemented and simulated efficient actor recovery protocol over wireless sensors and actor networks. The simulation is conducted on OMNET++ simulator. The size of the network is 1400 × 1400 square meters. Nodes are deployed randomly in the network. The main objective of simulation is to determine the performance of the proposed EAR algorithm in order to ensure the effectiveness of the protocol in presence of QoS parameters, energy efficiency when incident of node failure occurs. In addition, the performance of proposed EAR algorithm is compared with known similar type of schemes such as RNF, DPCRA, ACR, and ACRA.

RNF, DPCRA, ACR, and ACRA are state-of-the-art actor failure recovery algorithms. Detailed description was provided in Section 3. The proposed algorithms manages cut-vertex actor failure and recovery while they differ in their selection and objective obtained while recovery as given in Table 1. The similar parameters including properties have been used for testing purposes.

The simulation scenario consists of 400 nodes including 27–54 actor nodes and 173–356 sensor nodes with a transmission range of 70 m. The sensor/actor nodes are arbitrarily deployed in a mesh fashion. The initial energy of the actor nodes is set 20 J and sensor nodes have 4 J. The bandwidth of the actor node is 4 Mbps, and maximum power consumption of the sensor/actor node for receiving and transmitting the data is set to 13.5 Mw and 15.0 Mw respectively. Sensing and idle modes have 12.4 mW and 0.60 mW, respectively. The total simulation time is 36 min that is enough to determine the effectiveness of the proposed versus stat-of-the-art schemes. However, the simulation time could also be minimized or maximized, and the pause time is 20 s set to warm up the nodes before beginning of the simulation. The results demonstrate presented here are the average of 10 simulation runs. The simulation parameters are summed up in Table 2.

The simulation consists of three simulation scenarios that replicate the real wireless sensors and actor wireless sensor network environment. The obtained simulation results are equitably significant and indistinguishable to realistic tentative results.

- Scenario-I: Sensors-to-actor communications. In this scenario, the source nodes are set as the sensors, while the destination nodes are set as actors. The multiple connections are setup with one actor. Thus, the actor node acts as the sink of the communication. There is 86.5%:13.5% ration of sensor-to-actor, and 20% mobile sensor nodes are set. In this scenario, we used different sizes of the network; 1000 × 1000 m^2^, 1200 × 1200 m^2^ and 1400 × 1400 m^2^.
- Scenario-II: Actor-to-actor communications. In this scenario, the distance between the two actors is 300 m. The distance is covered by less than 4 hops. This scenario involves multi-hop communication among the actors. In this scenario, a maximum 54 actors are used.
- Scenario-III: Actor-to-sensor communications. In this scenario, communication is done between actors and sensor. The distance between actor and sensor is set to 250 m. The number of hops are 5 and mobility of the nodes is 20% in this communication. There is 13.5%:86.5% ration of sensor-to-actor, and 20% mobile sensor nodes are set. In this scenario, we used different sizes of the network; 1000 × 1000 m^2^, 1200 × 1200 m^2^ and 1400 × 1400 m^2^.

15–70 connections are set up among the nodes. The connections start working randomly during the warm up time. The source and destination nodes are randomly chosen in each scenario. Based on the simulation, we obtained interesting results including the following parameters:

- Number of alive Days.
- Residual Energy.
- Actor/Sensor Recovery time.
- Data Recovery.
- Time Complexity.

### 5.1. Number of Alive Days

**Figure 5:**
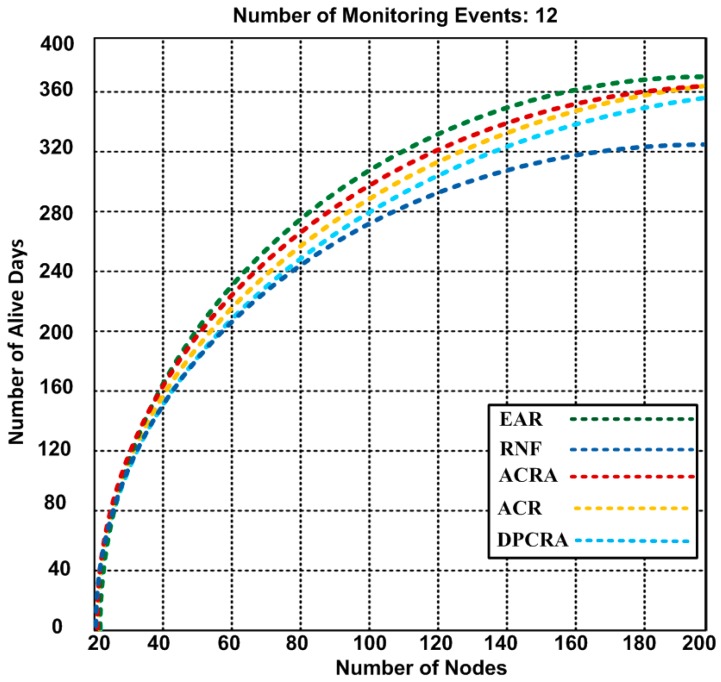
Number of alive nodes after completion of 12 events with 1200 × 1200 m^2^ network topology (Results obtained from Scenario-1).

**Figure 6:**
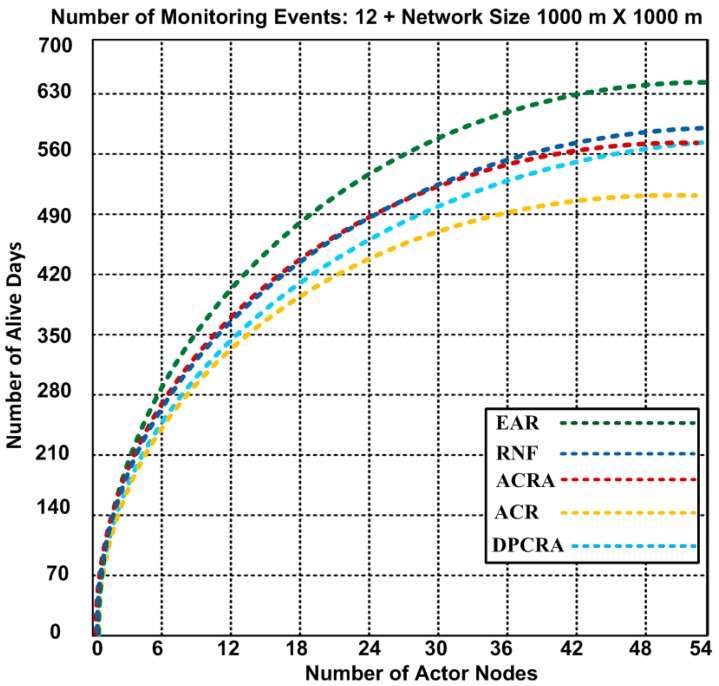
Number of alive nodes after completion of 12 events with 1000 × 1000 m^2^ network topology (Results obtained from Scenario-2).

**Figure 7:**
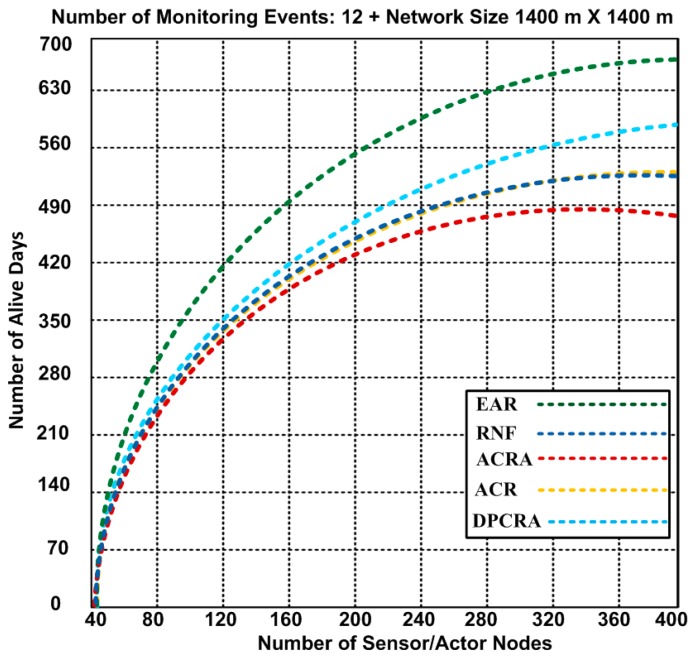
Number of alive nodes after completion of 12 events with 1400 × 1400 m^2^ network topology (Results obtained from Scenario-3).

Extended network lifetime has a significant role in improving the performance of the applications. In Figure 5, Figure 6 and Figure 7, the performance of EAR is shown and compared with RNF, DPCRA, ACR, and ACRA in form of number of alive nodes. In these experiments, we used the results of three scenarios with different network topologies. In scenario-1, we used 1200 × 1200 m^2^ network size with number of maximum 200 nodes that include 27 actor and 173 sensor nodes. Sixty five connections are established to cover the entire scenario. Based on the results, we observed that 24 nodes have 78 alive days in our approach and same days with compared approaches, but when the number of nodes increase up to maximum 200 nodes, then the number of alive days are different. Our approach has slight edge over other competing approaches. In our approach, the nodes are alive up to 367 days as compared with other approaches that have less alive days. RNF approach has 323 alive days, and ACRA has 362 alive days. In scenario-1, our proposed EAR has improvement over other competing approaches of 1.36–11.98%. In scenario-2, we used 1000 × 1000 m^2^ network size with number of maximum 54 actor nodes with 15 connections. Based on the results, we observed that actors are alive for 643 days in our approach, while other approaches have actor life of 512–592 days. ACR approach has less 512 alive days, and RNF has 588 alive days. In scenario-2, our proposed EAR has improvement over other competing approaches of 7.93–20.37%.

In scenario-3, we used 1400 × 1400 m^2^ network size with number of maximum 400 nodes that include 54 actor and 346 sensor nodes. Seventy five connections are established to cover the entire network scenario. Based on the results, we observed that nodes have lifetime 671 days in our proposed EAR approach, whereas other approaches have alive nodes 485–571 days. The performance of the network in ACRA is greatly affected which has minimum of 485 alive days. Therefore, our proposed EAR has improvement over other competing approaches of 14.9–27.71% in scenaroio-3.

The reason of the better stability of our approach is the usage of the RSSI model that helps to determine the proper distance between sensor-to-sensor, sensor-to-actor and actor-to-actor nodes. Furthermore, the network integration message process connects the entire network. As a result, bottlenecks are avoided. In case of the node failure, the backup node discovery process is initiated, that does not only improve the throughput, but also extends the nodes’ lifetime.

### 5.2. Residual Energy

**Figure 8:**
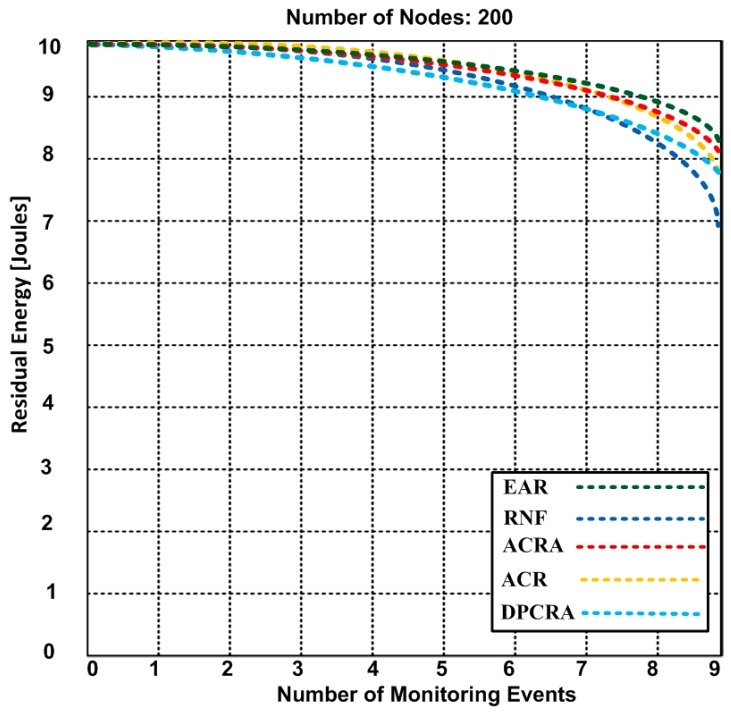
The residual energy of EAR and other competing approaches based on 9 event-monitoring.

**Figure 9:**
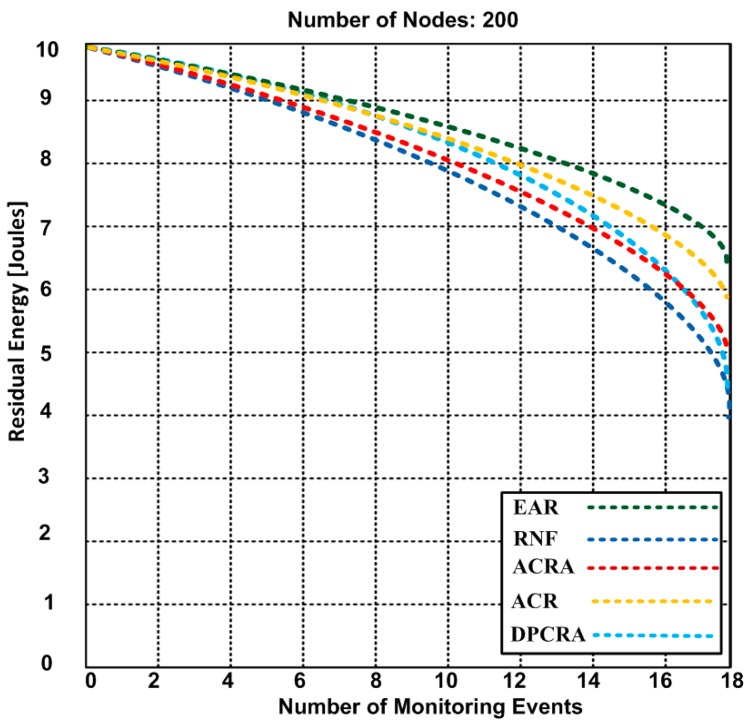
The residual energy of EAR and other competing approaches: RNF, DPCRA, ACR, and ACRA based on 18 event-monitoring.

**Figure 10:**
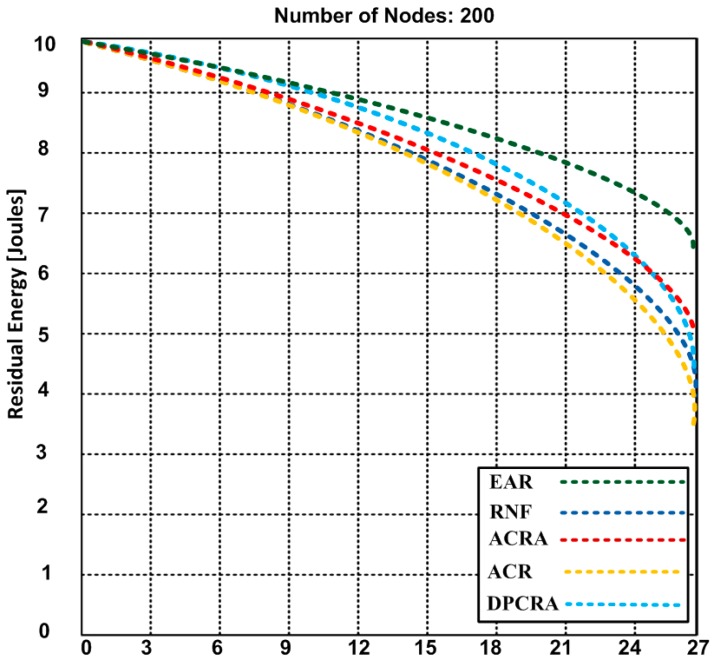
The residual energy of EAR and other competing approaches: RNF, DPCRA, ACR, and ACRA based on 27 event-monitoring.

The residual energy is the remaining energy level of the actor/sensor nodes when concluding the event(s). Here, we discuss an average residual energy level of the actor/sensor nodes after monitoring of different number of events. Figure 8, Figure 9 and Figure 10 compare the residual energy of EAR with those of the RNF, DPCRA, ACR, and ACRA at nine, 18 and 27 events respectively. The sensor/actor nodes have a higher residual energy with the EAR after completion of the events. In this experiment, the results are obtained based on three scenarios:

(14) $$E_{res}=\left[E_{in}-\;\left\{\frac{n*\left(P_{c}*E_{red}\right)+n*\left(E_{amp}*P_{c}\right)}{2E_{ra}}+r^{2}\;\left(N_{n}-1\right)\right\}+\;\left\{\frac{\left(\Delta C_{p}*E_{red}\right)+\left(E_{amp}*P_{c}\right)}{2E_{ra}}\right\}+\left\{\frac{{\left\{n*\left(P_{c}*E_{red}\right)+n*\left(E_{amp}*P_{c}\right)\right\}}^{2}}{2E_{ra}}+\;h^{2}\;\left(N_{n}-1\right)\right\}\right]\;$$

In Figure 8, 70 connections are established for nine events. The actor-to-actor are 12 connections, actor-to-sensor are 32 connections and sensor-to-sensor are 26 connections. Each connection consumes different energy. However, we obtained an average of overall residual energy for the entire network based on the number of connections. We observed in Figure 8 that the residual energy of our proposed approach has 8.4 J with nine events as compared with other approaches have residual energy ranging from 6.9–8.2 J. When we increased the events up to 18 in the Figure 9, the residual energy of our approach marginally dropped and became 7.4 J and competing approaches have residual energy from 4.2–5.9 J. In Figure 8 and Figure 9, RNF has less residual energy due to sending additional control message during the actor node failure process. In Figure 10, EAR has 6.7 J of residual energy whereas other competing approaches have 3.4–5.2 J residual energy. ACR has minimum residual energy when the number of events increase in Figure 10. The reason of the minimum residual energy is due to the decision tree that is incorporated in the reactive routing protocol. Our approach has higher residual energy for all events because our proposed model determines the forwarding capacity of each sensor/actor node prior to transmission which helps to avoid the node failure. The residual energy of sensor/actor is calculated using Equation (14) and the description of the used notations is given in Table 3.

### 5.3. Actor/Sensor Recovery Time

**Figure 11:**
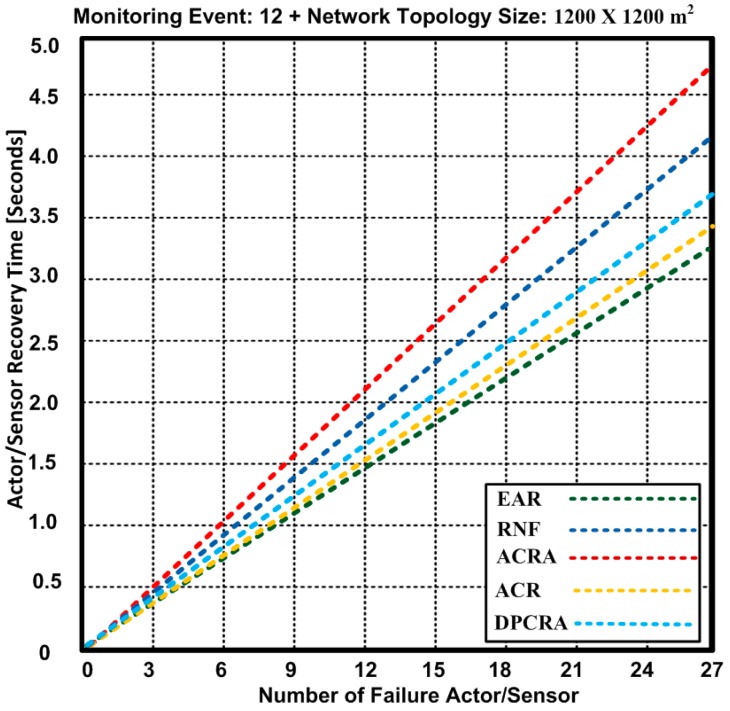
Number of failure actors/Sensors and required actor recovery time for EAR, RNF, DPCRA, ACR, and ACRA approaches with 1200 × 1200.

**Figure 12:**
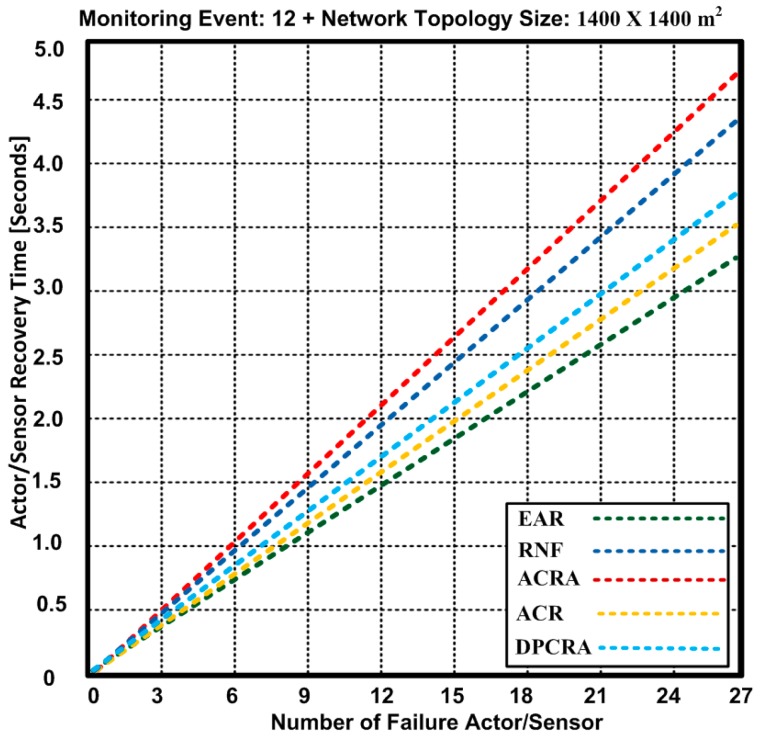
Number of failure actors/Sensors and required actor recovery time for EAR, RNF, DPCRA, ACR, and ACRA approaches with 1400 × 1400.

The actor recovery time is of high significance for network improvement and running applications on it. When the actor fails, then it is important to initiate the prompt recovery process to avoid the reduction in the network performance. Figure 11 and Figure 12 show the actor recovery time of the proposed EAR algorithm and other competing approaches: RNF, DPCRA, ACR, and ACRA. In these experiments, we used two different network topologies: 1200 × 1200 m^2^ and 1400 × 1400 m^2^. In Figure 11, we used 1200 × 1200 m^2^ network topology with 48 connections.

Based on the results, we observed that EAR has overall minimum actor/sensor recovery time. We determined an actor recovery time for maximum 27 failure nodes including 11 actors and 16 sensors nodes. At the maximum of 27 failure nodes, EAR has 3.25 s actor/sensor recovery time while other approaches have 3.6–4.7 s. The results show that EAR has 3.19–20% improvement over other competing approaches.

In Figure 12, we used 1400 × 1400 m^2^ network topology with 60 connections. Based on the results, we observed that EAR has overall minimum actor recovery time. We determined an actor/sensor recovery time for maximum 27 failure nodes including 11 actors and 16 sensors nodes. At the maximum 27 failure nodes, EAR has the same time of 3.25 s as obtained with 1200 × 1200 m^2^ network topology with 60 connections. It is confirmed that the increase in the network topology does not affect the actor/sensor recovery time while other approaches have 3.62–4.75 s. The result show that EAR has 3.21–20.8% improvement over other competing approaches.

The results confirm the soundness EAR in terms of an actor recovery time due to contention-free forwarding capacity of the nodes. In addition, particular RSSI value is selected for traffic forwarding process that makes the process of actor recovery much easier. As, all of the existing approaches either attempt to recover the failure actor or try to reduce the overhead, but our proposed approach reduces the power consumption and delivers the data without contention. Furthermore, it improves the backup node selection process in case of node failure or being disjoint. These characteristics of EAR help reduce the actor recovery time as compared with other approaches.

### 5.4. Data Recovery

Although data loss is very critical issue, very little information is publically released even when substantial data is lost. A wide variety of failures can cause physical mutilation to the quality of service of the applications. To retain the lost data, the backup recovery approaches perform vital role. However, data recovery methods are not capable enough particularly in wireless sensor and actor networks. In our proposed approach, we have a node monitoring algorithm that monitor the status of the node prior to failure as well as post-failure.

**Figure 13:**
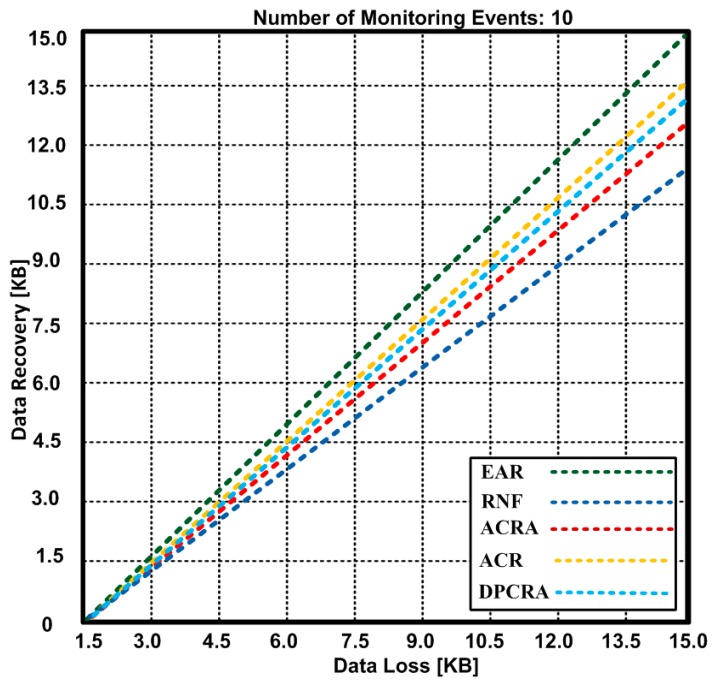
Data loss vs. Data recovery during 10 events.

**Figure 14:**
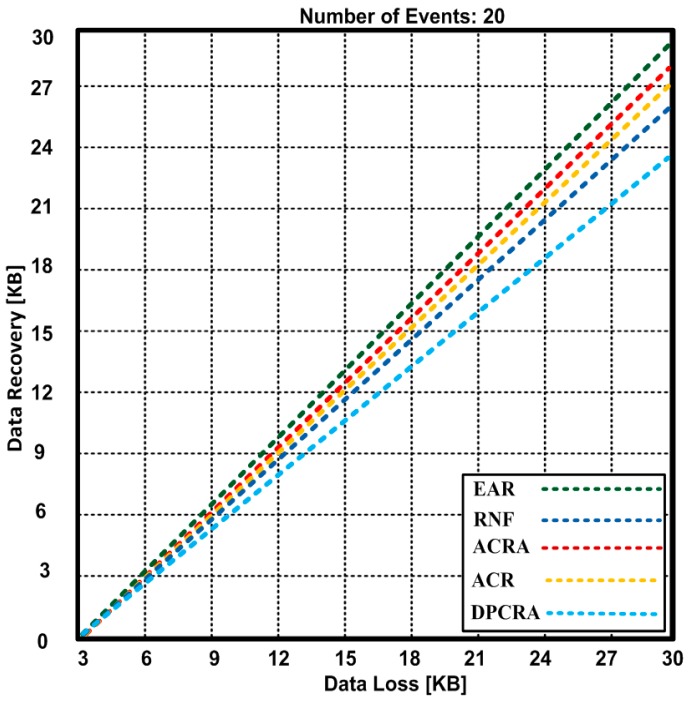
Data loss vs. Data recovery during 20 events.

As a result, backup nodes take the responsibility of storing the data. Figure 13 and Figure 14 show data loss and recovered data with 1400 × 1400 m^2^ network topology using 72 connections. In Figure 13, the total data loss is 15 KB when monitoring 10 events. Based on the results, we observed that EAR lost 15 KB data and recovered 15 KB that shows our scheme of data recovery is fault-tolerant, whereas other approaches also lost the same amount of data, but recovered 11.1–13.5 KB data. It is confirmed that EAR has 10%–26% improvement over other competing approaches.

In Figure 14, data loss is 30 KB with 20 events. As some of the events are not highly critical so that less amount of data is lost with 20 events. As EAR recovers 29.82 KB out of 30 KB that is quite better recovery as compared with other competing approaches. The other competing approaches are greatly affected due to the increase in events so that other approaches have recovery data from 23.8 to 29.1 KB. The least adaptable data recovery algorithm is DPCRA with 20 events. The results also validates that EAR has 2.41%–20.66% improvement over other competing approaches.

### 5.5. Time Complexity

**Figure 15:**
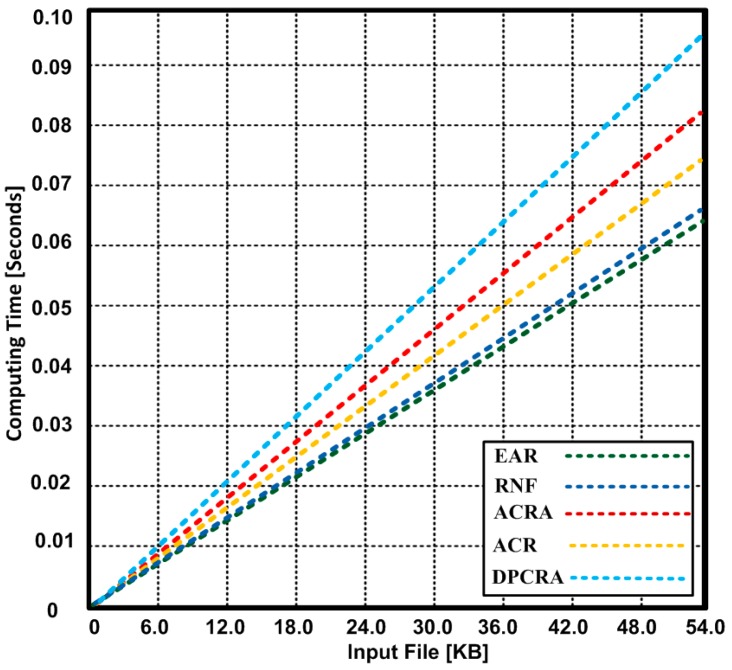
Execution Time of EAR, RNF, DPCRA, ACR, and SCRA in terms of seconds.

The quality of the running applications depends on the time complexity of algorithm. The time complexity is normally measured by calculating the number of basic operations and time consumed for those operations performed by the algorithm. The algorithm that takes less time improves the performance of the running applications. In Figure 15, we show the average time consumed for input data processing by EAR algorithm in comparison with RNF, ACRA, ACR and DPCRA. Based on the experimental results, we observed that EAR sent more input data in minimum time as compared with other competing algorithms. EAR sent maximum 54 KB input data within 0.065 s, whereas other protocols took 0.067–0.094 s in sending the same amount of data. EAR achieves minimum time because using the single operation for either pre-failure or post-failure recovery processes help reducing the time complexity.

To analyze the time complexity of EAR, lets determine the processes involved in the pre-failure and post failure process. In EAR, each critical actor node has a pre-assigned backup actor node which monitors its critical node. If consecutive heartbeat messages are not received from the critical node, backup actor node handles the recovery process. Let’s assume the critical actor is designated as (AC) and its backup node is designated (AB), the following instructions illustrate the pre-failure process: 

        {If (AB.HeartbeatMonitor(AC) == false);
        Ab.Recover(AC);
        }

While post failure process is handled by the backup node (AB). Thus, AB moves towards the failed actor (AC) location in order to recover the network partition. Also, the neighbors of the critical node will use the stored information to communicate with the backup node (AB) in order to restore connectivity.

        PostFailure(AC, AB) 
        { 
        Move(AB, c); 
        Connect(AB, Neighbors(AC)) 
        }

To calculate the time complexity for the previous operation, we assume that each process takes a time *T*(*n*) which is illustrated in Table 4. As shown in Table 4, each statement takes O(1).

**Figure 16:**
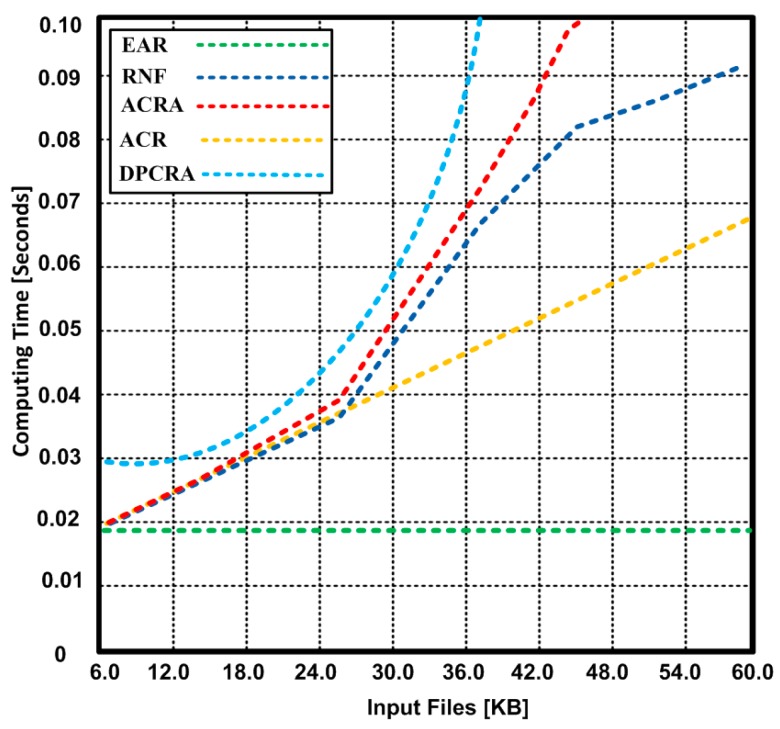
Big-O Complexity Chart.

Figure 16 is used to represent the time complexity analysis of different algorithms. It is used to illustrate the complexity description of the algorithms. The time complexity of EAR and other competing algorithms is obtained using Big O notation is given in Table 5.

### 5.6. Overall Performance of EAR

Based on the experimental results, we show the significance and improvement of EAR approach and comparison of other approaches in Table 6.

## 6. Conclusions

An Efficient Actor Recovery (EAR) algorithm is introduced in this paper. The approach is based on the Received Signal Strength (RSSI). Unlike most published approaches, EAR differentiates between critical and non-critical nodes and allocates a suitable backup node from its neighboring nodes, which is also chosen based on the signal strength and regulates the nodes in its surrounding locality. EAR consists of novel RSSI model that helps applying probability density function for finding the correct location of the sensor node. In addition, it shows the relationship between the received energy of the wireless signals and transmitted energy including the required distance among the actor-sensor nodes. Furthermore, EAR is supported by three algorithms for performing the network monitoring process, network integration and message forwarding process, and routing process for actor node to avoid the failure node. EAR approach has been validated using simulation of OMNET++ and compared with other known approaches; RNF, DPCRA, ACR, and ACRA. The experimental results demonstrate that EAR outperforms other competing approaches in terms of data recovery, number of alive days of the nodes, residual energy and data loss.

## Figures and Tables

**Table 1:** Compared Algorithms

Algorithm	Actor Deployment	Actor Recovery Selection	Aim
ACR	Random	FH selection based on node distance to failed actor	Actor recovery while Reduce total travel distance
RNF	Random	Neighbors containing smallest block	Limit path extend between nodes.
DPCRA	Random	FH handles the recovery based on smallest block of nodes	Minimum number of recovery nodes having their minimum travel distance
ACRA	Randomly	Actor node with high transmission power and higher coverage area is selected, and connectivity is recovered	Recover network from cut-vertex actor failure by limiting actor movement and using sensors as connecting bridges

**Table 2:** Summarized simulation parameters for the proposed EAR.

Used Parameters	Parameters’ Description
**Transmission Range**	70 m
**Sensing Range of sensor node**	35 m
**Initial energy of a sensor node**	4–10 J
**Initial energy of an actor node**	20–40 J
**Sensing Range of an actor node**	65 m
**Bandwidth of sensor node**	50 Kb/S
**Bandwidth of an actor node**	4 Mb/s
**Simulation time**	36 min
**Maximum nodes**	400
**Number of sensors**	173–346
**Static Sensor**	80%
**Mobile Sensor**	20%
**Number of actors**	27–54
**Actor-Sensor Ratio**	13.5:86.5
**Network Size**	1000 × 1000 m^2^, 200 × 1200 m^2^, 1400 × 1400 m^2^
**Number of hops in network**	18 Maximum
**Models**	EAR, RNF, DPCRA, ACR, and ACRA
**Buffering capacity at sensor and actor**	50 & 300 Packets buffering capacity at each sensor and actor respectively
**Mobility (Speed of the nodes)**	0 m/s to 12 m/s
**Data Packet size**	512 bytes
**Initial pause time**	20 s
**Rx energy**	12.4 mW
**Tx energy**	0.60 mW
**Power Intensity**	−14 dBm to 13 dBm.
**Total simulation time**	36 min

**Table 3:** Notations and descriptions.

Notations	Descriptions
n	Number of the packets
$P_{c}$	Control packets
$E_{in}$	Initial energy
$E_{res}$	Residual energy
$E_{red}$	Energy consumed for the radio signal
$E_{amp}$	Energy consumed for amplifying the signal
$E_{ra}$	Mean Energy consumed for amplifying the signal and radio
h	Number of hops
$N_{n}$	Number of sensor/actor nodes

**Table 4:** Pre-failure and post-failure time analysis of EAR using O Big operation.

Process	No. of Statements	Statement	Running Time	Time Complexity
**Pre-failure process**	1	If (AB.HeartbeatMonitor(AC) = false);	*T*(*n*) = 1	O(1)
	2	Ab.Recover(AC)	*T*(*n*) = 1	O(1)
**Post-Failure**	1	move(ab, ac)	*T*(*n*) = 1	O(1)
	2	Connect(AB, Neighbors(AC))	*T*(*n*) = 1	O(1)

**Table 5:** Time Complexity of EAR, RNF, DPCRA, ACR, and ACRA using O Big operation.

Name of Approaches	Excellent ()	Description
**EAR**	O(1)	Excellent
**RNF [10]**	O(log *n*)	Good
**ACRA [13]**	O(*n* log (*n*))	Bad
**ACR [7]**	O(*n*)	Fair
**DPCRA [11]**	O(2*n*)	Worst

**Table 6:** Improvement of EAR in percentile as compared with competing approaches: RNF, DPCRA, ACR, and ACRA.

Parameters	Improvement in EAR		
**Number of alive Days (Scenario-1)**	0.5–11%		
**Number of alive Days (Scenario-2)**	7.93–20.37%		
**Number of alive Days (Scenario-3)**	14.9–27.71%		
**Residual Energy in J at 9, 18, & 27 (Events)**	9	18	27
	2–15%	16–29%	16–33%
**Actor Recovery time (Seconds) with 1200 × 1200 m^2^ network topology**	3.19–20%		
**Actor Recovery time (Seconds) with 1400 × 1400 m^2^ network topology**	3.21–20.8%		
**Data Recovery of 15 KB with 10 Events**	10–26%		
**Data Recovery of 30 KB with 20 Events**	2.41–20.66%		
**Execution Time (Seconds)**	0.2–2.9%		
